# What’s Wrong with Evolutionary Causation?

**DOI:** 10.1007/s10441-020-09381-0

**Published:** 2020-04-13

**Authors:** Jan Baedke

**Affiliations:** Department of Philosophy I, Ruhr University Bochum, Universitätsstr. 150, 44801 Bochum, Germany

**Keywords:** Evolutionary causation, Agency, Individuality, Environment, Popper, Kuhn, Lakatos

## Abstract

This review essay reflects on recent discussions in evolutionary biology and philosophy of science on the central causes of evolution and the structure of causal explanations in evolutionary theory. In this debate, it has been argued that our view of evolutionary causation should be rethought by including more seriously developmental causes and causes of the individual acting organism. I use Tobias Uller’s and Kevin Laland’s volume *Evolutionary Causation* as well as recent reviews of it as a starting point to reflect on the causal role of agency, individuality, and the environment in evolution. In addition, I critically discuss classical philosophical frameworks of theory change (i.e. Popper’s, Kuhn’s and Lakatos’) used in this debate to understand changing views of evolutionary causation.

## Introduction

Causality and evolution are a problematic couple. In the last decades, biologists and philosophers of biology have increasingly questioned the standard view of evolutionary causation according to which the central causes of evolution are genetic variation (causing trait variation), inheritance (causing transmission of trait variation and resemblance between parents and offspring), and natural selection (causing adapted traits to spread in populations). This critical stance is spelled out in at least five different ways: It has been argued that (a) natural selection has no or merely little causal power in evolution (Goodwin 1994), (b) natural selection cannot explain all evolutionary relevant processes (Laland et al. 2015), (c) the ultimate-proximate distinction excludes developmental causes from evolutionary explanations (Laland et al. 2013), (d) natural selection is not a causal force working on the levels of populations (Sober 1984), but a statistical epiphenomenon resulting from the reproduction and survival of individuals (Matthen and Ariew 2002), and (e) not genes and populations but the developing and acting organism should be a starting point of evolutionary explanations (Laland et al. 2015; Walsh 2015).

In recent years, these theoretical discussions have coincided with a paradoxical situation in evolutionary biology, in which directly opposing causal explanations of the same developmental phenomena increasingly emerged (e.g., Laland et al. 2014; Wray et al. 2014; see Baedke et al. 2020). For instance, niche construction has been described as a causal starting point of evolutionary trajectories (Laland et al. 2005), or as nothing but an ‘extended phenotype’ (Scott-Phillips et al. 2014). Developmental plasticity has been explained as facilitating and directing evolutionary processes (reviewed in Sultan 2017; Uller et al. 2020) or as merely an adaptation to environmental stochasticity. In addition, epigenetic inheritance has been seen as an (partly) independent cause of variation (Jablonka 2017) and as being caused by genetic programs (Dickins and Rahman 2012). One might see these conflicts as hinting towards opposing causal frameworks underlying evolutionary biologists’ research practices and explanations.

This situation constitutes the theoretical and empirical background of Tobias Uller’s and Kevin Laland’s new volume *Evolutionary Causation* (MIT Press, 2019). It emerged from a workshop on ‘*Cause and Process in Evolution*’ held at the Konrad Lorenz Institute for Evolution and Cognition Research in 2017 (Baedke 2017). It contains 15 chapters by evolutionary biologists and philosophers of biology that offer important and novel perspectives on how different views of causation can bias, limit, enrich, or expand evolutionary theory, and thus affect our views on what phenomena require evolutionary explanations and how they should be explained. From the biological side, these contributions focus on topics such as the origin of variation and bias in variation (Stoltzfus, chapter 3; Moczek, chapter 4), environmental induction (Dayan et al. chapter 5), phenotypic plasticity and extra-genetic inheritance (Sultan, chapter 6; Watson and Thies, chapter 10), niche construction (Laland et al., chapter 7; Duckworth, chapter 8; Watson and Thies, chapter 10; Otsuka, chapter 12; Chiu, chapter 14), and evolutionary transitions from individuals to collectives (Helanterä and Uller, chapter 9; Watson and Thies, chapter 10). From the philosophical side, contributions discuss the suitable level of organization on which evolutionary relevant causes are located, i.e. the level of individuals or populations (Walsh, chapter 11), how certain ontological assumptions about the set of evolutionary relevant causes affect methodological decisions on how to study these causes (Otsuka, chapter 12), the role time scales play in accepting different slower or faster processes as evolutionary relevant ones (Pocheville, chapter 13), how niche construction through the organism’s experience of its environment (rather than its manipulation) affects natural selection (Chiu, chapter 14), and how biological information can be conceptualized and measured as a common causal factor of both development and evolution (Stotz, chapter 15).

These views on evolutionary causation are highly diverse and, at first sight, the reader might assume they are too diverse to be able to point towards a coherent novel causal framework for evolutionary biology. Some adopt compositional accounts, in which part-whole relations structure evolutionary relevant causal relations (Watson and Thies). Others highlight a scalar account, in which chosen time scales structure what counts (or should count) as a causal explanation (Duckworth, Pocheville). Others, explicitly or implicitly, develop their positions close to that of developmental systems theory (Oyama 1985; Oyama et al. 2001). This includes, for example, the idea of causal reciprocity (discussed in Pocheville). This view argues that instead of dividing biological causes in two classes, developmental proximate causes and ultimate causes like natural selection (Mayr 1961), both causes should rather be seen as forming causal feedback loops that permanently constitute one another, for example, through niche construction (Laland et al.; see Buskell 2019). Other chapters explore, in a ‘dialectical manner’, the causal relations between high degrees of variation (e.g., in phenotypic plasticity) with high degrees of stability and evolutionary stasis (e.g., highly conserved gene regulatory networks, robust ecological interactions; Moczek, Duckworth). However, besides this diversity on views about what qualifies as evolutionary causation, there are also similarities.

Other reviewers of this volume (Dickins 2020; Svensson 2020) have framed similarities between the chapters as emerging from the fact that most of the contributors (are said to) support recent calls for expanding evolutionary theory (see Pigliucci and Müller 2010a; Laland et al. 2015). Subsequently, these reviews critically discuss the historical narrative of how the view on evolutionary causation defended in the so-called ‘extended evolutionary synthesis’ (EES) differs from that of the modern synthesis. They also question whether the historical narrative about the limitations of the modern synthesis, widely discussed in the EES debate, is accurate. Here, I will try to avoid these issues as far as possible, especially because in this volume the central issues on causation have been decoupled most widely from questions about the EES. In most cases the EES has been simply ignored (for an exception, see Otsuka). Instead, in order to identify similarities in the causal views presented in this volume we might take a different approach: All authors seem to agree that the above position (a) – defended, for example, in biological structuralism – is a too radical view of evolutionary causation. Instead, most of them believe that our understanding of which causes should do the explaining in evolutionary theory needs to be rethought in more moderate ways along the lines of two arguments: argument (b-c), i.e. the inclusion of *developmental causes* into evolutionary explanations, and argument (d-e), i.e. the explanatory focus should lie on (or be expanded by) the *causes of the individual acting organism*.

The first argument, criticizing the “exclusion of development in evolutionary explanations” (Uller and Laland: 4), has been widely discussed before and is a cornerstone of many empirically and theoretically insightful chapters in this volume. Therefore, I will focus on some points related to the second argument that I think are thus far underrepresented in the current debate on evolutionary causation.

## Individuals, Agency and Environments

In many of the chapters the organism is attributed a number of important causal roles in evolutionary processes. Among others, the active organism establishes the robustness of ecological interactions through its plasticity and niche constructing behaviors (Duckworth), it mediates evolutionary transitions in individuality (Watson and Thies), and its experience of the environment modulates niche construction effects and changes natural selection from an external to a (at least in part) internally constructed cause (Chiu). Such an organism-centered perspective on evolutionary causation is tempting as it is seemingly in line with the many recent findings on how organisms affect the internal origin and transmission of variation and how they externally affect selection pressures. Nonetheless, to provide the organism a special explanatory status in evolutionary theory is also a quite challenging conceptual enterprise (see Baedke 2019). I want to highlight only three issues here related to this challenge. They refer to evolutionary individuality, agency, and the concept of environment.

If we consider organismic individual agents as the core entities that partake both in development and evolution, attempts to integrate the two realms have to show in each case that, in fact, it is the same unit that develops *and* evolves. In other words, if we want to unify development and evolution through the unit of the biological individual (being the one entity that partakes in both) this unit needs to meet criteria of both physiological (e.g., metabolic, immunological) and evolutionary individuality. Evolutionary individuals have been traditionally conceptualized as reproductive units with differential fitness and shared lineages, so-called ‘Darwinian individuals’ (Godfrey-Smith 2009), or as units of selection, so-called ‘interactors’ (see Hull 1980). Unfortunately, both of these physiological and evolutionary units do not always coincide (Godfrey-Smith 2013; Pradeu 2016). For example, some individuals (e.g., holobionts) form developmental but no reproductive units, as they include a multitude of lineages (e.g., microbial ones). Other possible units of selection (like genes or populations) are not identical with physiological individuals. Thus, a physiological individual may not necessarily be an evolutionary unit or vice versa.

Against this background, and against the discussions in this volume, it might become more fruitful for developmentalist views of evolutionary causation to explore different, less classical definitions of evolutionary individuality. Rather than trying to identify, first and foremost, units of selection or reproduction, the evolutionary relevant individual could be more generally construed as a causal nexus of intra-, inter- and extra-organismic processes (occurring on different developmental and evolutionary time scales) that can affect the origin, direction, and speed of evolutionary processes. This could include a range of different physiological individuals—developing organismic agents—that experience and interact with their environment in ways that affects the availability and character of variation and the organization of groups, populations and whole ecosystems in ways that might have evolutionary-relevant downstream effects. For example, these developmental units might causally contribute to the origin and stabilization of ‘Darwinian individuals’ at different levels of organization (as discussed in insightful chapters by Tobias and Helanterä as well as Watson and Thies). However, by no means must their evolutionary relevance be restricted to necessarily satisfying the above classical criteria of evolutionary individuality. In short, linking developmental with evolutionary causes through the unit of the (organismic) individual might necessitate approaching new and possibly broader ways to conceptualize evolutionary individuality, apart from reproductive and fit units.

This also includes clarifying what organismic agency actually should mean in a developmentally-informed causal framework of evolution. The present volume highlights ‘active phenotypes’ (Watson and Thies), ‘active agents’ and ‘purposive organisms’ (Laland et al.). Unfortunately, a consistent theory of agential causation that would strengthen especially the status of niche construction as a theory is missing. Attempts to provide such a framework (e.g., Laland et al.) draw on classical understandings of purposefulness of organisms through thermodynamics and self-organization (see Schrödinger 1944; see also Nicholson 2018; Baedke 2019). However, it remains unclear how this framework can incorporate those cases of purposeful behavior of organisms that include, for example, the experiential side of niche construction (Chiu; see also Sultan 2015). It should be able to flesh out the different causal roles the agent is performing in changing its environment (by modifying it) and by changing its relation to its environment (by experiencing it). There is an important analytical difference between the two that any theory of agency needs to incorporate, as both cases can have very different evolutionary effects.

This challenge might make necessary delving deeper into widely neglected issue of organismic teleology. According J.B.S. Haldane “Teleology is like a mistress to a biologist: he cannot live without her but he’s unwilling to be seen with her in public” (attributed to Haldane by Pittendrigh; Mayr 1988: 63). While some have begun to directly address the issue of teleology and how it could be linked to evolutionary causation (e.g., Walsh 2015), in the present volume this difficult task is mostly left untouched. Earlier proposals for organism-centered views of evolution that have tried to develop a non-vitalist framework of organismic goal-directedness might be used as a stepping stone for such an enterprise (see Haldane 1917; Schaxel 1919; Russell 1924, 1945; Bertalanffy 1928). Against longstanding anti-teleological traditions in evolutionary thought, these teleological and constructionist views argued, in line with more recent approaches, that the organism purposefully molds itself and its environment in development and evolution, like “clay modeling itself” (Russell 1924: 61). However, these approaches should be also seen as a historical warning sign. They were never able to develop enough argumentative persuasiveness, possibly due to their lack of formalization, in order to advance into mainstream evolutionary reasoning.

Another related issue concerns the concept of environment. The volume’s contributions explore in detail various forms of organism-environment and genotype-environment relationships, but what the environment itself is and how it should be causally explained is only addressed in passing (but see Moczek, Chiu). The authors usually adopt a causal framework which, due to the dominant agential status of organisms, makes them reject externalist perspectives of the selective environment (see Moczek 2015). As Waddington (1959: 1636) described this view: “Natural selection is far from being as external a force as the conventional picture might lead one at first sight to believe.” In this standard view, the environment is conceptualized as a “source of error that reduces precision in genetically studies,” and thus one has “to reduce it as much as possible” (Falconer 1960: 140). In contrast, according to the internalist or constructionist perspective, organisms (co-)construct their environments and, as a consequence, themselves. Rather than adjusting their traits to suit their ‘external’ local environments, organisms alter their environments in a flexible manner so that these environments suit their traits. Thus, the causal realm of the environment is no longer constituted ‘from within’ but through the organismic agent.

While this view has become widely accepted in developmentalist accounts to evolutionary causation, it lacks clarity. What is needed is a classification of the different ‘environments’ identified in evolutionary research. These include, among others, collective or individual, general or unique, homo- or heterogeneous, invariant or spatio-temporally flexible, selective or constructed, passive or actively generative, external or internal, and experienced or ‘acted on’ environments. Which disciplinary or experimental settings in evolutionary biology favors certain of these views of the environment and why? Which views of the environment do recent trends towards organism-environment reciprocity and individual environments favor, and why? Furthermore, what are the epistemological and methodological challenges going along with each of these concepts? These and related conceptual questions should be addressed head-on to support first attempts to empirically fleshing-out the character of different environments, like organism-constructed environments (Clark et al. 2020). This will also allow clarifying the different causal roles the environment plays in evolution as well as identifying the problems different views on the environment pose for experimental setups and explanatory standards in evolutionary research. The present lack of conceptual clarity may lead to ambiguities about the boundaries of environments. What counts as environment in one field may be understood as (part of) an organism in another. In addition, this situation can contribute to poor communication and impossibility of collaboration across fields.

## Beyond Popper, Kuhn and Lakatos

Another question this volume triggers is whether the views on evolutionary causation presented here impose some kind of theory change to the field (see Dickins 2020, Svensson 2020). Theory change has been one of the most debated topics in the history of philosophy of science. In the 20th century it was a central topic of works by authors like Karl Popper, Thomas Kuhn and Imre Lakatos. According to these models, scientific change occurs through falsification of theories (Popper), through revolutionary breaks with past theories (Kuhn), or, as a middle ground between the two, though modifications of research programs (with a hard core and protective belt of auxiliary hypotheses) that have strategies to protect themselves from falsification (Lakatos). Even though these approaches largely focused on addressing cases of scientific change in physics and mathematics, and have problems in application on biology, they had quite an impact on how biologists reasoned about change in their own field (see Mayr 1976, 1982; Lewontin et al. 1984). And while philosophy of science has long moved on from these rather simplistic and in many ways problematic views of scientific change from the early and mid-20th century, many biologists today are still quite happy with discussing them. They are widely applied to understand whether or not the causal framework provided by evolutionary theory is currently changing (see Dickins and Rahman 2012; Tanghe et al. 2018, Laland et al., Otsuka; for discussion, see Fábregas-Tejeda and Vergara-Silva 2018).

Let us have a closer look at one of these attempts by Dickins (2020), and at what views of current evolutionary theory the above philosophical approaches lead to. By drawing on Lakatos, Dickins argues that the best confirmed and thus best protected theoretical core of evolutionary theory (described by the modern synthesis) is the theory of natural selection, flanked by other explanations of how populations change (e.g., through drift). This core is the only legitimate starting point to develop models that lead to a better understanding of evolutionary phenomena:

What natural selection does is enable the construction of falsifiable hypotheses about particular biological systems. As such, the MS [modern synthesis] might be seen as a viable research program, following Lakatos. (Dickins 2020: 513)

In contrast, developmentalist views of evolution—including their different causal frameworks like reciprocal causation—are given a different status. According to Dickins, they merely address feedback effects that should be studied first and foremost in ecology, not evolution. They cannot be included into the theoretical core of evolutionary theory, because these theories on the origin of variation, developmental bias, inclusive inheritance and niche construction, do actually not challenge natural selection directly. In contrast, “these challenges are really around quibbles with regard to specific models” (ibid.). Thus, following Lakatos and Popper, they address and try to falsify auxiliary hypothesis derived from the theoretical core. In other words, they ask for different constrains and other degrees of abstraction in models of natural selection by more strongly considering developmental processes. Dickins adds:

Nonetheless the MS should welcome a fully worked theory of the emergence of the phenotype, of variation, and of inheritance and so all of this work needs to be considered. What is not presented is a real challenge to the core axioms of the MS. […] My suspicion is that true and productive challenges to evolutionary biology will arise from efforts directed toward the origins of life itself, and constraints upon this afforded by physics. (Dickins 2020: 513)

Two problems should be highlighted here: First, why, at all, should a critique of evolutionary biology only be considered a valid contender or ‘real challenge’ if it criticizes (or tries to falsify) the theory of natural selection? In order to meet this high bar, the current developmentalist framework would basically have to defend a position similar to that of biological structuralism (see point (a) above), which failed in changing evolutionary theory in the 1980s–1990s largely because it argued for a too radical theory of evolution without natural selection. This shows that, if we assume that natural selection forms (large part of) the theoretical core of a Lakatosian research program in evolutionary biology, this automatically constrains the conditions under which evolutionary theory could be changed. This assumption can easily distort understandings of how the current debate affects and possibly reshapes evolutionary theory. Second, why should only more fundamental sciences like physics and investigations of more causally up-stream events like the origin of life, be able to address the core of evolutionary theory? This only makes sense within a view of evolutionary theory that rests on the existence of certain “hard-core axioms” (ibid.). However, axiomatic reasoning can hardly be considered adequate for explanatory practices in biology; nor is any dogmatic stance that sees natural selection as no longer in need of proof within biology (see, e.g., Mayr 2002: 26).

As we have seen, when starting from classical philosophical frameworks of theory change, there is a danger of introducing stereotypical, axiomatic (maybe even dogmatic) and oversimplifying views of theory change to the current evolutionary debate. However, this danger exists for both critics and defenders of developmentalist approaches to evolutionary causation. Currently, the debate seems somewhat trapped within a theoretical framework that understands theory change to happen within a spectrum between normal science and revolutions, with more or less radical changes in research programs. This limitation is at least in part due to philosophers of science who so far have largely failed to provide scientists in the field with more accurate conceptual frameworks that could help them reasoning about and framing opposing views of evolutionary explanations. In other words, philosophers have failed to enrich these biological debates through fruitful active engagement (of the form requested by Pigliucci in his chapter). This should certainly not mean that there exists no theory change in the field. However, this issue is not related to questions about falsifications, revolutions, and axiomatic cores of theories.

What could an alternative approach look like? What developmentalist explanations of evolution are thought to provide is a usually neglected causal perspective that should broaden our understanding of evolution (Uller and Laland; see also Laland et al. 2015; Uller et al. 2020). Adding explanations of developmental causes, like developmental bias, phenotypic plasticity, niche construction, and inclusive inheritance, to the causal picture of evolutionary theory should lead to “more complete explanations” (Laland et al. 2015) and a “significantly expanded explanatory capacity” (Pigliucci and Müller 2010b: 12). In short, these explanations should increase the explanatory power of evolutionary theory. What exactly does this mean? What we learned from the debate so far is that explanatory power seems to be not (or not directly) linked to how much an explanation is supported by empirical evidence. In fact, while there is often agreement in evolutionary biology over the existence of these developmental phenomena (Laland et al. 2014; Wray et al. 2014; Svensson 2020; Dickins 2020), at the same time, their explanatory relevance is questioned (Wray et al. 2014; Futuyma 2017; Svensson 2020; Dickins 2020). In short, developmental causes are accepted, but their explanatory power is not.

Against this background, one has to understand due to which explanatory virtues developmentalist explanations are better, and which tradeoffs between explanatory standards—like precision (specificity), proportionality, sensitivity, and idealization—those accounts face that seek to integrate developmentalist and genetic explanations of evolution (Baedke et al. 2020). In other words, this perspective highlights a narrative according to which evolutionary theory, expanded by developmental causes, currently faces tensions between different explanatory standards. New explanations are accepted or rejected based on criteria of explanatory power entrenched in the field. If developmentalist explanations do not meet these criteria (like a specific degree of precision, sensitivity or proportionality) scientists are skeptical whether they carry explanatory power and increase our understanding of evolution. In such cases, the integration of developmentalist and populationist views within a more pluralist framework of evolutionary causation, as requested by many authors of this volume (e.g., Pigliucci, Dayan et al., Watson and Thies, Walsh, Otsuka), is rejected by critics. In addition, this perspective on explanatory power highlights the necessity of evolutionary biologists to stop reasoning about theoretical cores, falsifiability and about whether we currently witness a gradual or revolutionary theoretical change in the field. Instead, they have to start jointly reflecting on which explanatory standards they want their evolutionary explanations and models to hold in the future.

## Conclusions

*Evolutionary Causation* provides an excellent collection of empirical and theoretical papers. They will surely work as stimulating starting points for both biologists and philosophers to reflect on what we consider to qualify as a cause and causal explanation in evolutionary theory. This is despite—or perhaps because—of the fact that the views presented do not form a coherent whole. Interestingly, this positive assessment is shared by more critical readers of this volume, who, at the same time, take the views presented to be largely in agreement with standard views of evolutionary causation and thus to be not that new after all (Dickins 2020; Svensson 2020). This might be due to a general reluctance of critiques to rework conceptual frameworks with proven quality or to rethink entrenched explanatory standards. But this might also be because the causal picture presented by advocates of developmentalist perspectives still lacks argumentative strength and persuasiveness (e.g., when it comes to agency, individuality and the environment). One thing is for sure. Both sides don’t do the debate a service in forcing it into simplistic frameworks of theory change. These approaches cannot do justice to the diversity of views of causation we currently witness in evolutionary biology. And they cannot provide guidance towards research practices that successfully integrate opposing views and explanatory standards within one research framework.
